# Cosmetic Breast Augmentation with Autologous Ex Vivo-Expanded Adipose-Derived Mesenchymal Stem/Stromal Cell (Stemform®)-Enriched Fat Grafts: A Study of the First Twenty-Two Real-World Patients

**DOI:** 10.1007/s00266-023-03711-6

**Published:** 2023-12-04

**Authors:** Frederik Penzien Wainer Mamsen, Anne Fischer-Nielsen, Jesper Dyrendom Svalgaard, Jesper Due Jensen, Bo Jønsson, Dominik Duscher, Josef Christensen, Michiel Van Leeuwen, Claes Hannibal Kiilerich, Laura Roider, Aris Sterodimas, Lea Munthe-Fog, Stig-Frederik Trojahn Kølle

**Affiliations:** 1 StemMedical A/S, Gyngemose Parkvej 50, 2860 Copenhagen, Denmark; 2 Department of Plastic Surgery, Aleris Hospitals, Gyngemose Parkvej 66, 2860 Copenhagen, Denmark; 3 Eberhard Karls University Tübingen, 72076 Tübingen, Germany; 4 Academic Stem Cell Center Vienna, Liechtensteinstrasse 96, 1090 Vienna, Austria; 5 Saint Louis University School of Medicine, 1008 S Spring Ave Suite 1500, St. Louis, MO 63110 USA; 6 IASO General Hospital, 15562 Athens, Greece; 7 CeriX Hospital, Strandvejen 191, 2900 Copenhagen, Denmark

**Keywords:** Breast augmentation, Lipofilling, Stemform, StemMedical, Adipose-derived mesenchymal stem/stromal cells, Ex vivo cell expansion, Fat graft, Graft retention

## Abstract

**Background:**

Fat grafting is commonly utilized in breast surgery, and since it was first described, clinicians and researchers have stridden towards improvement of graft retention. Current advancements include adding adipose-derived mesenchymal stem/stromal cells (MSC(AT)s), which have demonstrated promise for improved graft retention.

**Objectives:**

This study reports outcomes for the first twenty-two patients undergoing breast augmentation (Stemform BA) or artificial implant replacement (Stemform AIR) with MSC(AT)-enriched fat in a real-world setting.

**Methods:**

Autologous MSC(AT)s were isolated and expanded ex vivo, then mixed with lipoaspirate and injected as enriched fat for Stemform BA and AIR. The breast volume was measured preoperatively and at 3 and 12 months postoperative using a 3D Infinity Dual-Lens Camera and LifeVizApp software. Additionally, independent plastic surgeons evaluated clinical images, and patient satisfaction was obtained at equal time points.

**Results:**

Twenty-two patients were included. All completed 3 and 12 months clinical follow-up and 3 months volume measurements. Nineteen patients completed 12 months volume measurements. The median fat graft retention at 12 months was 95.7% (IQR = 82.44–103.12%) for Stemform BA patients and 113.0% (IQR = 94.8–131.2%) for Stemform AIR patients. The Stemform BA patients had a median breast enlargement of 172.0% (IQR = 156.7–241.0%). The implant replacement volume of Stemform AIR patients was 102% (IQR = 85.1–130.3%). The patient reported 92.8% and 100% would elect to repeat treatment if they had the opportunity for Stemform BA and Stemform AIR, respectively.

**Conclusion:**

Breast augmentation and breast implant replacement patients receiving ex vivo-expanded MSC(AT)-enriched fat grafts had high graft retention and patient satisfaction scores. The paper confirms the clinical efficacy of using ex vivo-expanded MSC(AT)s.

*Level of Evidence V* This journal requires that authors assign a level of evidence to each article. For a full description of these Evidence-Based Medicine ratings, please refer to the Table of Contents or the online Instructions to Authors www.springer.com/00266.

## Introduction

With low complication rate, fat grafting is increasingly used as a natural filler for cosmetic breast augmentations. This allows patients to avoid an unnatural augmented appearance and the complications associated with breast implants [1]. Since its origin, fat grafting for breast augmentation has been greatly refined [2–4], but outcomes remain unpredictable due to volume resorption with retention rates between 20 and 75% [5–7]. A recent advancement in fat grafting is the addition of regenerative cells found in the stromal vascular fraction (SVF) [8] with Dr. Yoshimura Kotaro being the first to apply regenerative cells in this setting [9]. The clinical effect of SVF on fat graft retention is still inconclusive, as most studies have not found consistent improvement in graft retention [10]. SVF is composed of a heterogenic cell population with 10–40% being adipose-derived mesenchymal stem/stromal cells (MSC(AT)s), which have the greatest regenerative potential [2, 11, 12]. A previously published randomized controlled trial (RCT) investigating MSC(AT)-enriched fat grafts for breast augmentation reported a median graft retention of 80.2% (IQR = 66.1–124.2%) when the fat graft was enriched with > 20 × 10^6^ MSC(AT)s/mL compared to a graft retention of 45.1% (IQR = 36.5–50.7%) in patients receiving conventional fat grafting [13].

This study investigates graft retention, patient-reported satisfaction, and outcome assessment by independent plastic surgeons for the first twenty-two patients who underwent ex vivo-expanded MSC(AT)-enriched fat grafting for breast augmentation (Stemform BA) or following artificial implant removal (Stemform AIR) in a real-world setting. The Stemform fat graft contains a minimum of [20 × 10^6^ homogeneous MSC(AT)/mL of fat] which is unreachable for other single-stage autologous cell solutions like SVF.

## Materials and Methods

### Protocol

The Stemform® product is for cosmetic use and as such the manufacturing and clinical use (mixing with autologous fat for lipoinjection, Stemform Procedure), is approved and regulated by the Danish Patient Safety Authority (DPSA) and authorized by a Tissue Establishment License. Data were collected for patients undergoing breast augmentation performed with MSC(AT)-enriched fat at Aleris Hospital, Copenhagen, Denmark, and CeriX Private Hospital, Copenhagen, Denmark, between 01 Jan 2020 and 01 Aug 2022. For all patients, StemMedical A/S provided the Stemform product, consisting of isolated and ex vivo-expanded autologous MSC(AT)s, which were subsequently mixed into the patient’s own fat during the procedure. Complete details on the handling of MSC(AT)s are described in the study by Kølle et al. [13]. However, in brief, freshly harvested lipoaspirate is washed in lactated Ringer’s and enzymatically digested by collagenase (GIDzymer-2 GMP Grade Collagenase by GID Bio), followed by centrifugation for 10 min at 600 g. The obtained SVF is then seeded at a density of 2500–5000 SVF cells per cm^2^ in cell factories and cultivated under hypoxic conditions for 14–21 days.

For each patient 100–150 mL of lipoaspirate was collected and processed as described above [13]. Two to three weeks later, the patients underwent liposuction of the thighs, lower back, abdomen and additional areas if desired, using a 4-mm cannula and processed with either a Revolve™ or TissuTrans™ device, in accordance with the manufacturer´s manual and with suction pressure of ≤ 40 kPa. The harvested volume varied based on desired augmentation which was determined preoperatively by the patient with surgeon guidance. A maximum volume increase of 400 mL per breast was enforced.

Following liposuction, the freshly harvested and processed lipoaspirate was mixed with the previously expanded MSC(AT)s by removing the fat syringe plunger and injecting the MSC(AT)s using a 14-gauge cannula while constantly moving. This was followed by gentle stirring until a uniform colour and consistency was achieved. For Stemform BA patients, the MSC(AT)-enriched fat was injected structurally through three to four small punctures using a 14-gauge injector cannula. In the Stemform AIR population, the MSC(AT) enriched fat was injected using tactile feedback as the surgeon felt the tip of the cannula by accessing the breast pocket through the incision used to remove the implant allowing precise injection in tissue planes.

All handling of the tissues and cells, including testing, procurement, processing, and distribution, was performed in accordance with standards in the Danish Tissue Act (implementation of the EU Tissues and Cells Directive) in a tissue establishment licensed by The Danish Patient Safety Authority.

### Volume Measurement with 3D Imaging

All patients had 3D breast scans using QuantifiCare—3D-Infinity dual lens camera prior to surgery and at 3 and 12 months post-operatively. With the patients in an upright position, three images were obtained and using the LifeVizApp software by QuantifiCare a 3D animation of the patient was generated. Following QuantifiCare guidelines, the “3D Track” feature was used to estimate the patient’s breast volume after placement of reference points, ensuring the breast footprint was covered. The lateral boarder was determined by the axillary end of the inframammary fold, and the medial boarder was determined by the sternal end of the inframammary fold. The upper pole of the breast footprint was determined from a lateral view at the transition between chest wall and the protrusion of the breast. The number of reference points varied depending on the breast size and shape. All images were analysed, according to the manufacturer’s manual in LifeVizApp software, by physicians who previously underwent training at the QuantifiCare headquarters in France and completed additional online training courses.

### Patient-Reported Satisfaction

A five-point scale (Table 1) was used to assess patient satisfaction with the breast appearance three months following augmentation procedure. The following prompt was used: “How satisfied are you with the appearance of your breasts?”.

**Table 1:** Satisfaction scale

5	Very satisfied
4	Satisfied
3	Neither satisfied/unsatisfied
2	Unsatisfied
1	Very unsatisfied

### Plastic Surgeon Imaging Assessment

In addition to evaluation of 3D scans and patient satisfaction, before and after photographs were evaluated on a five-point scale (Table 1) by board certified plastic surgeons.

Clinical images of 6 total patients from the Stemform BA subgroup were collected, including 2 patients each with the highest, median, and lowest total graft retention. Similarly, the patient with the highest, median, and lowest total graft retention was identified from the Stemform AIR subgroup, totalling 3 patients. Prior to distribution all clinical images were retouched to remove distinct characteristics, such as freckles, birthmarks, and tattoos, and lighting was corrected to aid visual comparability. No changes were made to beautify or enhance surgical results. Images of the nine selected patients (6 BA and 3 AIR) were then distributed to 3 board-certified plastic surgeons in different countries, the USA (Atrium Health Wake Forest, North Carolina), Austria (TF-Plastic Surgery, Vienna), and Germany (BG Klinik Tübingen). Each of the 3 selected plastic surgeons then distributed the images to plastic surgery colleagues for evaluation, thus ensuring independent and nonbiased assessments.

The independently reviewing surgeons who received the pre- and post-augmentation images were asked to rate the results on a five-point scale (Table 1). The question presented with each set of images was: “How satisfied would you be with the result of the volume retention given that you injected the volume of fat figured below in a single augmentation procedure?” The only information presented to the surgeons was injected fat volume and size of the removed implants if relevant. An example of representative pre- and postsurgical images is demonstrated in Table 2. The full collection of clinical images assessed by the independent plastic surgeons can be found in “Appendix” with one being replaced due to withdrawal of consent.

**Table 2:** Clinical images of breast augmentation

Patient 1: Before	Patient 1: After
Patient 1: Before side	Patient 1: After side
Pre-Volume = 189 mL | Injected = 358 mL [24 × 10^6^ MSC(AT)s/ml fat]	Post-Volume = 506 mL | Fat Retention = 89% BMI stable (0.0 points)
Patient 2: Before	Patient 2: After
Patient 2: Before side	Patient 2: After side
Pre-Volume = 194 mL | Injected = 314 mL [26 × 10^6^ MSC(AT)s/ml fat]	Post-Volume = 432 mL | Fat Retention = 76% BMI decrease of 1.2 points
Patient 3: Before	Patient 3: After
Patient 3: Before side	Patient 3: After side
Pre-Volume = 78 mL **| **Implant volume = 320 cc **| **Injected = 300 ml [21 × 10^6^ MSC(AT)s/ml fat]	Post-Volume = 391 mL | Fat Retention = 105% BMI decrease of 0.5 points
Patient 4: Before	Patient 4: After
Patient 4: Before side	Patient 4: After side

The injected volume is presented as the average fat graft volume for each patient's two breasts. The retention rate is the average fat retention of the two breasts subtracted from the implant volume

To ensure an unbiased assessment, the reviewing surgeons sent the evaluation to the respective plastic surgeon who initially distributed the image file. All communication with the reviewing surgeons was through the three selected distributing plastic surgeons, no direct communication was performed by the research team. Data were then structured in Microsoft Excel and plotted in Prism Graph Pad 9. Unless specified otherwise, all data below have been presented as medians with interquartile ranges.

## Results

### Patient Demographics and Breast Volume

Twenty-two patients were included in this real-world patient study. All patients completed the procedure and the three months follow-up. Nineteen patients completed the 1-year follow-up. Two patients did not show up for control measurements and one patient had other cosmetic breast surgery between three months and 1-year follow-up and was therefore excluded.

#### Stemform BA Patients

**Fig. 1:**
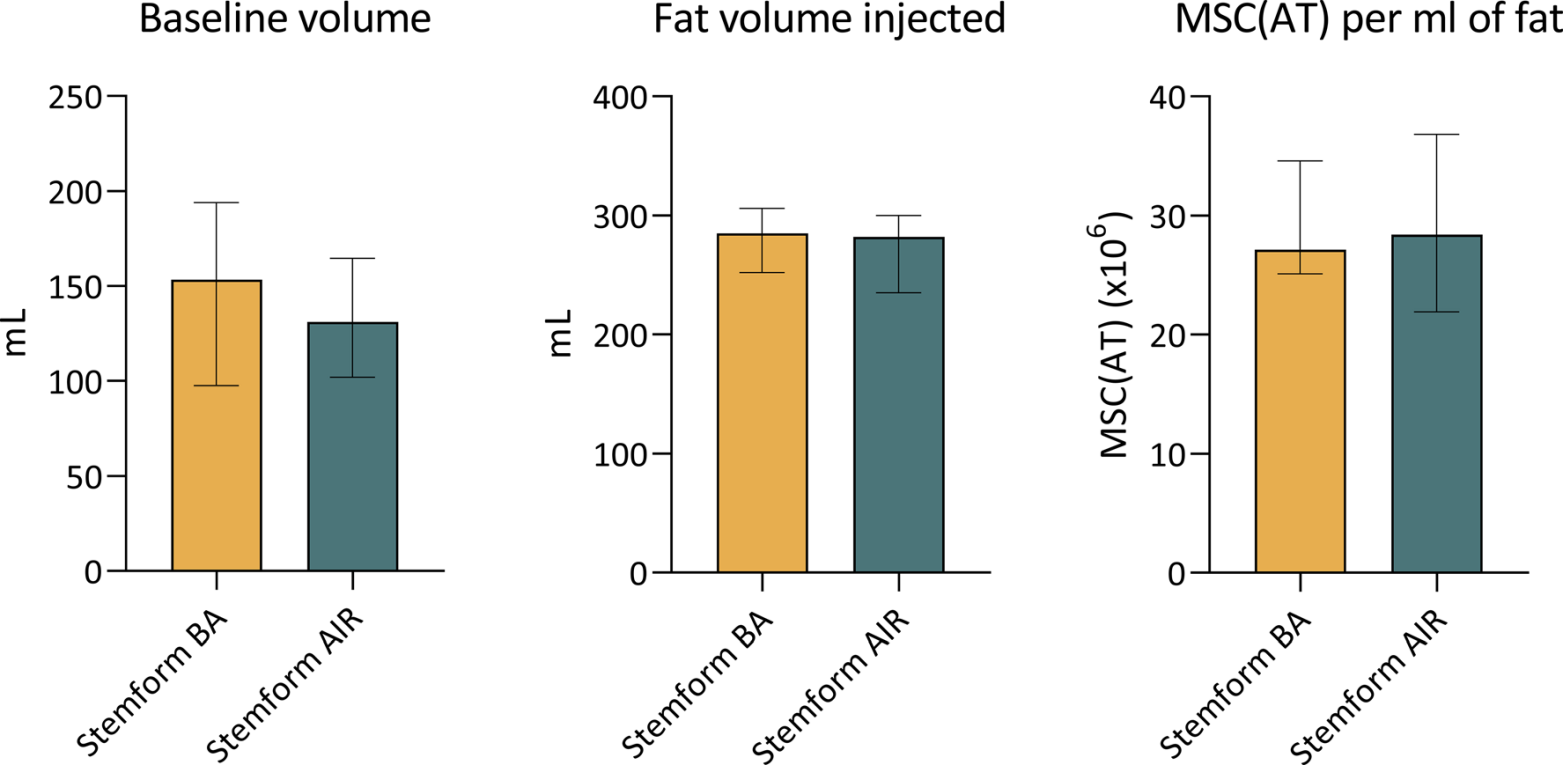
Grafting data. The graphs are presented with median values and interquartile ranges

**Fig. 2:**
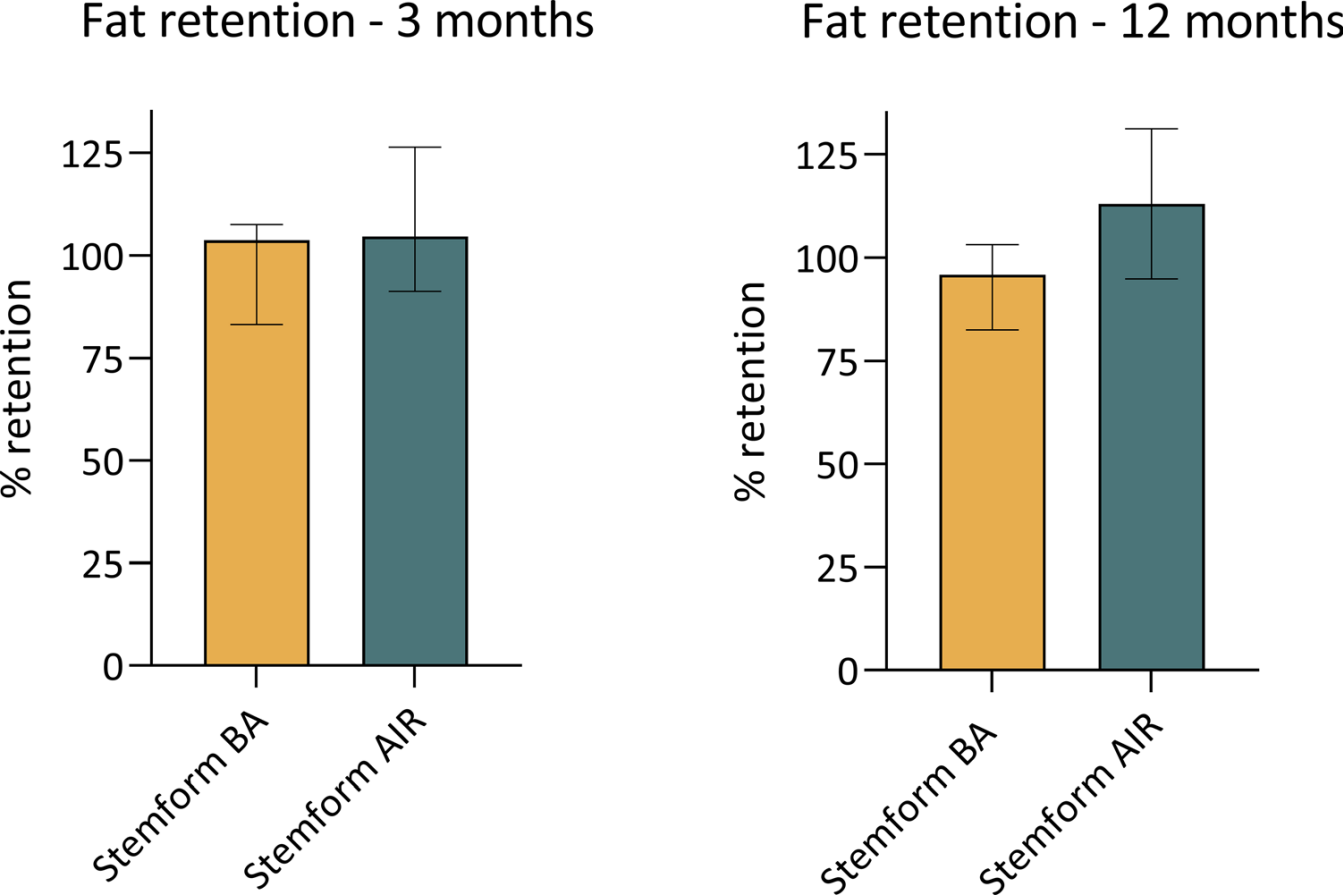
Graft retention rates. The graphs are presented with median values and interquartile ranges

The 17 patients who received Stemform BA had a median baseline breast volume of 154 mL (IQR = 98–194 mL) (Fig. 1A). The median injected volume of fat was 285 mL per side (IQR = 252–306 mL) (Fig. 1B) enriched with a median of 30 × 10^6^ (IQR = 25–36 × 10^6^) MSC(AT)s per mL of fat (Fig. 1C). Three months after treatment, the patients had a median fat graft retention of 104% (IQR = 83–108%) (Fig. 2A). Twelve months after the treatment the patients had a median fat retention of 96% (IQR = 82–103%) (Fig. 2B). The median BMI was calculated at 21.1 (IQR = 20.3–22.5), 21.6 (IQR = 20.5–23.3), and 20.8 (IQR = 20.3–21.6) for pre-operatively, 3 months follow-up, and 12 months follow-up, respectively. The calculated breast enlargement factor (the percentage of breast enlargement compared to initial breast volume) was 172% (IQR = 157–241%).

Fourteen of the seventeen patients completed the three months questionnaire, evaluating the satisfaction of pre- and post-operative breast appearance. The mean baseline score was 2.6 (SD ± 1.0) on a scale from 1 to 5. Post-augmentation this increased to a median of 4.0 (SD ± 0.92). 92.8% of the patients would elect to repeat treatment if they had the opportunity. Neither cell dose, injection volume, nor retention rate correlated with improved patient satisfaction.

Ten independent plastic surgeons evaluated the patient images before and after the procedure (Table 2 and “Appendix”) and scored satisfaction with the volume retention on a 1–5 scale (Table 1). The mean score was 4.1 (SD ± 0.60) at 3 months post-op.

#### Stemform AIR Patients

Five patients had breast implants and underwent implant removal and subsequent breasts enlargement with enriched fat grafting using the Stemform product (Stemform AIR). To obtain the baseline volume measurements for this subset of patients, the preoperative scan was performed, and the implant volume was subsequently subtracted. Stemform AIR patients had a median baseline breast volume of 131 mL (IQR = 102–165 mL) (Fig. 1A), and their median breast implant volume was 288 mL (IQR = 210–338 mL). The median injected fat volume was 282 mL (IQR = 235–300 mL) (Fig. 1B) enriched with a median of 28 × 10^6^ (IQR = 22–37 × 10^6^) MSC(AT)s per mL of fat (Fig. 1C). Three months after the treatment the patients had a median fat graft retention of 105% (IQR = 91–126%) (Fig. 2A). Twelve months after the treatment the patients had a median fat retention of 113% (IQR = 95–131%) (Fig. 2B). The median BMI was calculated to 20.1 (IQR = 19.8–22.8), 21.0 (IQR = 20.1–22.6), and 20.7 (IQR = 20.0–24.6) for pre, 3 months follow-up, and 12 months follow-up, respectively. The measured implant replacement factor (the percentage of implant volume substituted with fat) was 102% (IQR = 85–130%).

All five patients completed the three months questionnaire, evaluating the satisfaction of pre- and post-operative breast appearance. The mean baseline score was 2.8 (SD ± 0.99) on a scale from 1 to 5. Post-augmentation this increased to a median of 4.0 (SD ± 0.63). 100% of patients would elect to repeat treatment if they had the opportunity. Neither cell dose, injection volume, nor retention rate correlated with improved patient satisfaction.

Ten independent plastic surgeons evaluated the patient images before and after the procedure (Table 2 and “Appendix”) and scored satisfaction with the volume retention on a 1–5 scale (Table 1). The mean score was 3.9 (SD ± 0.78) at 3 months post-op.

## Discussion

In this study, we evaluated outcomes from a case series of twenty-two patients receiving autologous breast augmentation or artificial implant replacement with enriched fat grafting using Stemform®, an MSC(AT)-based product. The cohort represents the results after a decade of optimizing the conditions and procedures related to liposuction, MSC(AT) isolation, cell culture, harvest, postharvest handling, mixing, and injection. The results presented are in accordance with previously published studies on fat grafts enriched with ex vivo-expanded MSC(AT)s, including two previously published RCTs [13, 15, 16]. In 2013, Kølle et al. were the first RCT published on human fat grafts enriched with expanded MSC(AT)s prior to injection. The study was conducted in an experimental setting with bolus injection and showed a mean graft retention of 80.9% in patients receiving MSC(AT)-enriched fat compared to 16% in the controls who received conventional fat grafting [15]. Another study published by Kølle et al. in 2020 demonstrated a median of 80.2% total graft retention in patients treated with the Stemform product compared to 45.1% in those treated with conventional lipofilling [13]. In contrast to the above-mentioned studies and the results reported in our case series, one clinical study from 2022 found no differences in fat graft retention between conventional lipofilling and grafts enriched with expanded MSC(AT)s [17]. We anticipate that these findings are due to differences in cell source, culturing conditions, cell doses, and cell handling throughout the process, which are all factors that could influence the retention outcome.

Graft-to-capacity ratio is defined as the volume of grafted fat relative to the volume of the recipient site. According to a study by Del Vecchio et al, a graft-to-capacity ratio greater than 117% (SD ± 22%) of the initial breast volume dramatically reduces fat graft retention [18]. In this study, the median breast enlargement factor was 172%. Considering that our reported IQR graft to capacity injections ranged from 157 to 241% of the initial breast volume, theoretically this patient cohort should have suboptimal fat graft retention [19].

The reason for some patients having higher than 100% fat graft retention is likely due to multiple factors. One being the MSC(AT) enrichment, others being metabolic status [20], weight changes and statistical variation in the 3D measurements [21]. The Stemform AIR patients had a higher retention rate than the Stemform BA patients; however, the group consisted of few patients. One could expect that the retention rate after implant removal would be lower due to injection spill into the dead space created by removal of the breast implant. To avoid this, the surgeons aided precise injection by feeling the tip of the injector cannula by accessing the breast pocket through the incision used to remove the implant. This may have generated a more precise distribution of the grafts. Another theory is that the pressure inside the breast pocket is lower due to the already expanded breast tissue due to the implant, thereby minimizing the increase in interstitial pressure of the recipient tissue and thereof collapse of capillaries supplying the area of the fat graft.

Results indicate that MSC(AT)-enriched fat grafts are a great surgical option for patients with a desire for natural looking autologous breast augmentation for both patient groups.

On average, the patients in this cohort reported improved satisfaction with their breasts by a median of 2 points (on a scale from 1 to 5). Of patients, 92.8% and 100% of Stemform BA and Stemform AIR reported that they would proceed with repeating the procedure if they had the opportunity. Diaz et al. investigated patient satisfaction after undergoing breast augmentation procedures with implants through BREAST Q. A total of 494 patients were included, of whom 86% reported being satisfied with their results after an average of six months [22]. The overall patient-reported satisfaction appears to be similar in terms of cosmetic/visual outcomes when comparing breast augmentation with artificial implants versus MSC(AT)-enriched fat grafting.

The patients in this study population who were not satisfied with the procedure outcome reported dissatisfaction due to topographical irregularities after liposuction and the naturally sloped shape of the breasts. Patients frequently commented that they were pleased with the increased breast volume but had expected the volume distribution to provide better upper pole fullness. This outlines the importance that physicians educate and emphasize that even with a high fat retention rate, the breast shape will still look natural and currently the most reliable way to obtain upper pole fullness is with an implant. With this patient feedback, our injection technique has been adjusted by increasing the volume injected in the upper poles and segments of the breast. Of note, at recent post-operative follow-ups, some improvement in upper pole fullness has been observed. Additional surgeon observations pertaining to improving patient selection and satisfaction were as follows:

1. Utilization of implants to determine desired breast volume during preoperative consultation may lead to unrealistic expectations. The upper pole projection created by the implant shells does not resemble the look of a breast that has been augmented or reconstructed using fat, thus distorting the patient expectation.
2. Postoperative swelling was interpreted as grafted volume by some patients. As the postoperative breast volume is higher than the grafted amount due to swelling, some patients may interpret it as a loss of fat graft rather than a passing side effect of the procedure. However, this has also been described in patients undergoing traditional augmentation with breast implants. In a study by Brown, 137 patients underwent breast augmentations with implants. When assessed at 12 weeks post-operatively 19.4% wished the implants had been larger, even though they trialled implant shells before surgery [23].

Although not directly reviewed with patients, another aspect of the patient-reported satisfaction score revolved around well-known side effects of liposuction, such as topographical irregularities and prolonged sensory alterations. It must be emphasized that due to the cosmetic nature of this augmentation procedure, the surgeon must leave the patient with perfect donor sites and utilize the required liposuction for fat harvest as a body sculpting opportunity and cosmetic procedure, thereby providing the patient the advertised benefit of two-in-one. The BMI range in this study was below average between 20 and 22.9, corresponding to only a modest to thin layer of subcutaneous fat, making it challenging to acquire the required fat volume for breast augmentation. In thin patients additional attention must be paid to avoid asymmetry, topographical irregularities, and loose skin resulting in poor cosmesis.

Current volume prediction and assessment methods in fat grafting are unmodified from the artificial implant field despite major differences between these two procedures. This presents a challenge for both patients and surgeons when managing outcome expectations, as grafting outcomes vary regardless of whether the fat graft is enriched with MSC(AT), SVF or others. We strongly advise fellow plastic and reconstructive surgeons to clearly communicate expected fat graft retention rates (25–80% for conventional fat grafts and 60–110% for MSC(AT)-enriched fat grafts) during pre-operative consultation. From our experience, pre-operative patient education regarding fat graft retention variability can improve the patient’s decision making and expectations, ultimately leading to fewer misunderstandings and high patient satisfaction.

## Conclusion

The first 22 patients receiving Stemform BA or Stemform AIR had a high fat retention rate, high patient-reported satisfaction with the breast appearance and high clinical satisfaction scores from independent plastic surgeons. The paper confirms the clinical efficacy of using a high concentration of ex vivo-expanded MSC(AT)s. New patient data will continuously be collected and published to report both long-term safety and efficacy of the procedure. Additionally, new ways to customize graft density are being pursued to improve the possible outcomes for patients seeking implant-like results providing both a predictable volume and shape.

## Appendix: Collection of clinical images assessed by independent plastic surgeons

Clinical images of breast augmentation with Stemform®

**Table:**

Patient 1: Before	Patient 1: After
Patient 1: Before side	Patient 1: After side
Pre-Volume = 189 mL | Injected = 358 mL [24 × 10^6^ MSC(AT)s/ml fat]	Post-Volume = 506 mL | Fat Retention = 89% BMI stable (0.0 points)
Patient 2: Before	Patient 2: After
Patient 2: Before side	Patient 2: After side
Pre-Volume = 95 mL | Injected = 194 mL [40 × 10^6^ MSC(AT)s/ml fat]	Post-Volume = 259 mL | Fat Retention = 84% BMI increase of 1.2 points
Fat graft retention rates ~ 110%	
Patient 3: Before	Patient 3: After
Patient 3: Before side	Patient 3: After side
Pre-Volume = 132 mL **|** Injected = 220 mL [26 × 10^6^ MSC(AT)s/ml fat]	Post-Volume = 372 mL **|** Fat Retention = 109% BMI stable (0.0 points)
Patient 4: Before	Patient 4: After
Patient 4: Before side	Patient 4: After side
Pre-Volume = 173 mL | Injected = 300 mL [26 × 10^6^ MSC(AT)s/ml fat]	Post-Volume = 495 mL | Fat Retention = 107% BMI increase of 0.8 points
Fat graft retention rates ~ 70–80%	
Patient 5: Before	Patient 5: After
Patient 5: Before side	Patient 5: After side
Pre-Volume = 89 mL **|** Injected = 250 mL [23 × 10^6^ MSC(AT)s/ml fat]	Post-Volume = 284 mL | Fat Retention = 78% BMI decrease of 1.5 points
Patient 6: Before	Patient 6: After
Patient 6: Before side	Patient 6: After side
Pre-Volume = 194 mL | Injected = 314 mL [26 × 10^6^ MSC(AT)s/ml fat]	Post-Volume = 432 mL | Fat Retention = 76% BMI decrease of 1.2 points
The injected volume presented as the average fat graft volume for each patient's two breasts	

Clinical images of artificial implant replacement (AIR) with Stemform®

**Table:**

Above 100% fat graft retention	
Patient 7: Before	Patient 7: After
Patient 7: Before side	Patient 7: After side
Pre-Volume = 78 mL **|** Implant volume = 320 cc **|** Injected = 300 ml [21 × 10^6^ MSC(AT)s/ml fat]	Post-Volume = 391 mL | Fat Retention = 105% BMI decrease of 0.5 points
Above 110% fat graft retention	
Patient 8: Before	Patient 8: After
Patient 8: Before side	Patient 8: After side
Pre-Volume = 127 mL **|** Implant volume = 288 cc **|** Injected = 282 mL [28 × 10^6^ MSC(AT)s/ml fat]	Post-Volume = 445 mL | Fat Retention = 113% BMI stable (0.0 points)
Lowest fat graft retention	
Patient 9: Before	Patient 9: After
Patient 9: Before side	Patient 9: After side
Pre-Volume = 131 mL | Implant volume = 250 cc | Injected = 300 mL [23 × 10^6^ MSC(AT)s/ml fat]	Post-Volume = 390 mL | Fat Retention = 86% BMI stable (0.0 points)
Representative pre- and postsurgical images distributed to reviewing plastic surgeons. The injected volume is presented as the average fat graft volume for each patient's two breasts. The retention rate is the average fat retention of the two breasts subtracted from the implant volume	
