# Correction: Cosmetic Breast Augmentation with Autologous Ex Vivo-Expanded Adipose-Derived Mesenchymal Stem/Stromal Cell (Stemform®)-Enriched Fat Grafts: A Study of the First Twenty-Two Real-World Patients

**DOI:** 10.1007/s00266-023-03798-x

**Published:** 2023-12-21

**Authors:** Frederik Penzien Wainer Mamsen, Anne Fischer-Nielsen, Jesper Dyrendom Svalgaard, Jesper Due Jensen, Bo Jønsson, Dominik Duscher, Josef Christensen, Michiel Van Leeuwen, Claes Hannibal Kiilerich, Laura Roider, Aris Sterodimas, Lea Munthe-Fog, Stig-Frederik Trojahn Kølle

**Affiliations:** 1 StemMedical A/S, Gyngemose Parkvej 50, 2860 Copenhagen, Denmark; 2 Department of Plastic Surgery, Aleris Hospitals, Gyngemose Parkvej 66, 2860 Copenhagen, Denmark; 3 Eberhard Karls University Tübingen, 72076 Tübingen, Germany; 4 Academic Stem Cell Center Vienna, Liechtensteinstrasse 96, 1090 Vienna, Austria; 5 Saint Louis University School of Medicine, 1008 S Spring Ave Suite 1500, St. Louis, MO 63110 USA; 6 IASO General Hospital, 15562 Athens, Greece; 7 CeriX Hospital, Strandvejen 191, 2900 Copenhagen, Denmark

**Correction: Aesthetic Plastic Surgery** 10.1007/s00266-023-03711-6

The original online version of this article was revised to correct the images in the Appendix labeled as Before and After for Patient 1.

The original article has been corrected.

